# Diagnostic capacities and treatment practices on implantation mycoses: Results from the 2022 WHO global online survey

**DOI:** 10.1371/journal.pntd.0011443

**Published:** 2023-06-28

**Authors:** Barbara Milani, Daniel Argaw Dagne, Hye Lynn Choi, Marco Schito, Heather Anne Stone

**Affiliations:** 1 WHO consultant, World Health Organization, Geneva, Switzeland; 2 Department of Control of Neglected Tropical Diseases [WHO/NTD], World Health Organization, Geneva, Switzeland; 3 Department of Regulation and Prequalification [WHO/RPQ], World Health Organization, Geneva, Switzeland; 4 CURE Drug Repurposing Collaboratory, Critical Path Institute, Tucson, Arizona, United States of America; 5 Office of Medical Policy, Center for Drug Evaluation and Research, United States Food and Drug Administration, Silver Spring, Maryland, United States of America; Albert Einstein College of Medicine, UNITED STATES

## Abstract

Between January and March 2022, WHO conducted a global online survey to collect data on diagnostic capacities and treatment practices in different settings for four implantation mycoses: eumycetoma, actinomycetoma, cutaneous sporotrichosis and chromoblastomycosis. The survey investigated the type of diagnostic methods available in countries at various health system levels (tertiary, secondary, primary level) and the medicines used to treat implantation mycoses, with a view to understanding the level of drug repurposing for treatment of these diseases. 142 respondents from 47 countries, including all continents, contributed data: 60% were from middle-income countries, with 59% working at the tertiary level of the health system and 30% at the secondary level. The results presented in this article provide information on the current diagnostic capacity and treatment trends for both pharmacological and non-pharmacological interventions. In addition, the survey provides insight on refractory case rates, as well as other challenges, such as availability and affordability of medicines, especially in middle-income countries. Although the study has limitations, the survey-collected data confirms that drug repurposing is occurring for all four surveyed implantation mycoses. The implementation of an openly accessible global and/or a national treatment registry for implantation mycoses could contribute to address the gaps in epidemiological information and collect valuable observational data to inform treatment guidelines and clinical research.

## Author summary

In June 2022, the World Health Organization (WHO) launched a framework for integrated control and management of skin-related neglected tropical diseases. The framework describes how mycetoma, chromoblastomycosis and other (deep) implantation mycoses including sporotrichosis should be addressed and integrated in the primary health care settings. Between January and March 2022, WHO conducted a global online survey to collect information on the medicines used to treat implantation mycoses worldwide. The survey was designed as part of a WHO collaborative project with the United States Food and Drug Administration to identify priority disease areas for pilot testing in CURE ID, a web-based application to help clinicians share their experiences in managing difficult-to-treat infectious diseases and thereby inform clinical research needs to support drug repurposing. The survey also explored the type of diagnostic methods employed in countries within different health care settings (tertiary, secondary, primary level) and the medicines used to treat implantation mycoses, to obtain a better understanding of the practices in diagnostic services and level of drug repurposing for treatment of these diseases.

## Introduction

Implantation mycoses (also referred to as deep mycoses) include a heterogeneous group of fungal diseases (and one bacterial infection) that develop at the site of transcutaneous trauma. Some implantation mycoses involve muscles, fascia, cartilage and bones, beyond the skin and the subcutaneous tissues.

The WHO global online survey on implantation mycoses was conceived as part of a WHO collaborative project with the United States Food and Drug Administration (US FDA) to identify priority disease areas for pilot testing of CURE ID. CURE ID is a web and mobile-based application, developed as a collaboration between the US FDA and the National Center for Advancing Translational Sciences, a part of the US National Institutes of Health (US NCATS/NIH), to help clinicians share their experiences in managing difficult-to-treat infectious diseases (including neglected tropical diseases) and thereby inform clinical research needs to support repurposing approved drugs for new clinical uses [1,2]. The survey was also supported by the CURE Drug Repurposing Collaboratory (CDRC), a public-private partnership established to advance the CURE ID platform, inform clinical research, and sharing the clinical evaluation of drugs used for indications not on their label.

The survey was designed to contribute relevant data to the WHO efforts to improve epidemiological surveillance, as well as diagnostic and treatment management of this group of diseases, which were included in the WHO list of Neglected Tropical Diseases (NTDs), starting with mycetoma in 2016 with a disease-specific World Health Assembly resolution [3]. Mycetoma and chromoblastomycosis were recognized and officially classified as neglected tropical diseases in 2016 and 2017, respectively [4]. Sporotrichosis, although not officially listed as a neglected tropical disease, was included in the road map for neglected tropical diseases 2021–2030, published by WHO in 2020, as part of the other deep mycoses [5].

The 2021–2030 NTD road map sets global targets and milestones to prevent, control, eliminate or eradicate 20 diseases and disease groups in alignment with the Sustainable Development Goals. The first target set for this group of diseases relates to improving or instituting surveillance. For example, only 1 out of 30 countries affected by mycetoma had a national control programme and surveillance system in place as of 2020. Among the actions identified in the road map, several related to the development of point-of-care diagnostic tests to improve diagnostic capacity, as well as availability of treatment regimens with improved efficacy, and design of shorter and higher efficacy regimens for mycetoma and chromoblastomycosis. Treatment efficacy with itraconazole is reported to be very low for eumycetoma (<30%) [5,6]. This survey further contributes to the understanding of treatment and programmatic challenges for this group of diseases. The results presented in this article shall also be considered in light of the WHO integrated control and management of skin NTD framework, launched in June 2022, where integrated interventions have been identified to be adopted by endemic countries [7].

## Methods

The WHO survey was conceptualized using a mix of information sources including published literature on diagnostic and treatment methods, as well as key informant interviews. An initial inquiry was conducted with 12 key informants (international and national experts on the subject matter) to investigate diagnostic and clinical practices, particularly in middle-income countries. Further, the survey preparation relied on a 2019 article co-authored by more than 20 experts on implantation mycoses for the diagnostic methods [8].

The draft survey questions were reviewed and improved with feedback provided by WHO, the US FDA, the CURE Drug Repurposing Collaboratory (CDRC), and external WHO advisors for neglected tropical diseases. The survey was designed using multiple-choice options (for example, pre-defined options were provided for diagnostic methods, pharmacological treatment, and non-pharmacological interventions). An option was available for participants to provide additional entries and comments for every question.

The survey was created using the WHO official survey tool (WHO Dataform). It was opened on 7 January 2022 and closed on 15 March 2022. To reach the widest possible audience and reduce language barriers, the survey was made available in three UN official languages: English, French and Spanish.

The survey was disseminated extensively through different channels including: key informants of the initial enquiry; global and regional associations on dermatology, mycology and NTDs; working groups on implantation mycoses; and social media. On 17 February 2022, a webinar was organized in collaboration with the International Society for Neglected Tropical Diseases (ISNTD), to launch and further disseminate the survey to more participants [9].

The survey analysis was partly generated by the WHO official survey tool for yes/no and multiple-choice answers. The free text options and comments were considered, aggregated, and analysed separately. Part of the data analysis, such as visual display of maps, was elaborated from the Excel Master File generated by the survey tool. The preliminary analysis was shared and feedback from WHO, the US FDA, the CDRC and external WHO advisors and experts on neglected tropical diseases was incorporated.

The survey was conceptualized to primarily collect qualitative information on the type of diagnostic methods available in the participant setting, the medicines used for treatment, the type of non-pharmacological interventions and the presence of refractory cases. A question was also included on affordability and availability of medicines in the participant setting to investigate if these had an impact on the selection and use of medicines, and hence on prescribers’ practice, and drug repurposing. The survey provided a qualitative indication on all these aspects, which should be considered, in light of the number and profile of respondents. The survey did not collect information on individual treatment regimens and treatment outcomes.

## Results

### Respondents’ profile: Country, setting and role

**Fig 1:**
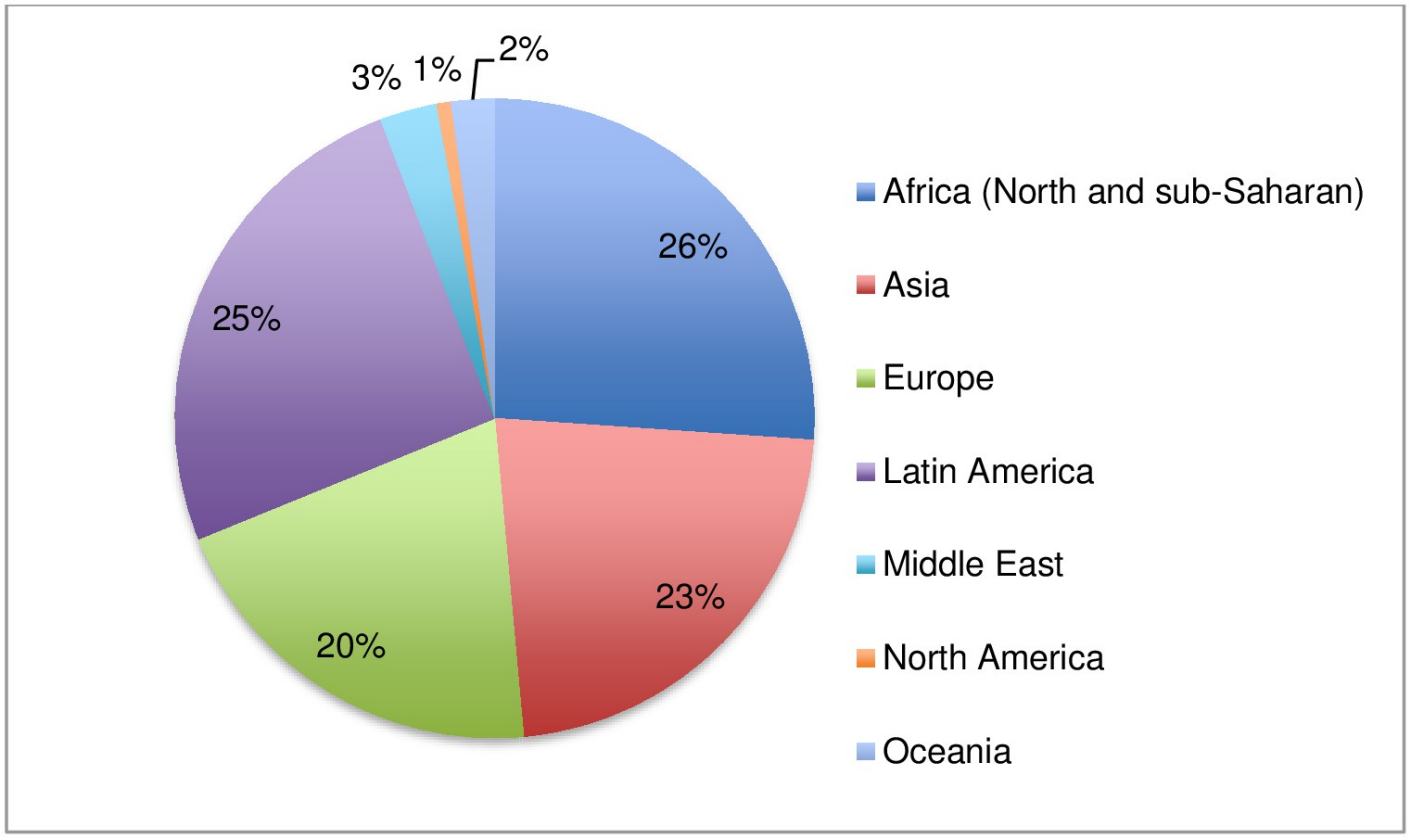
Indication of geographical distribution of respondents by continent/region.

**Fig 2:**
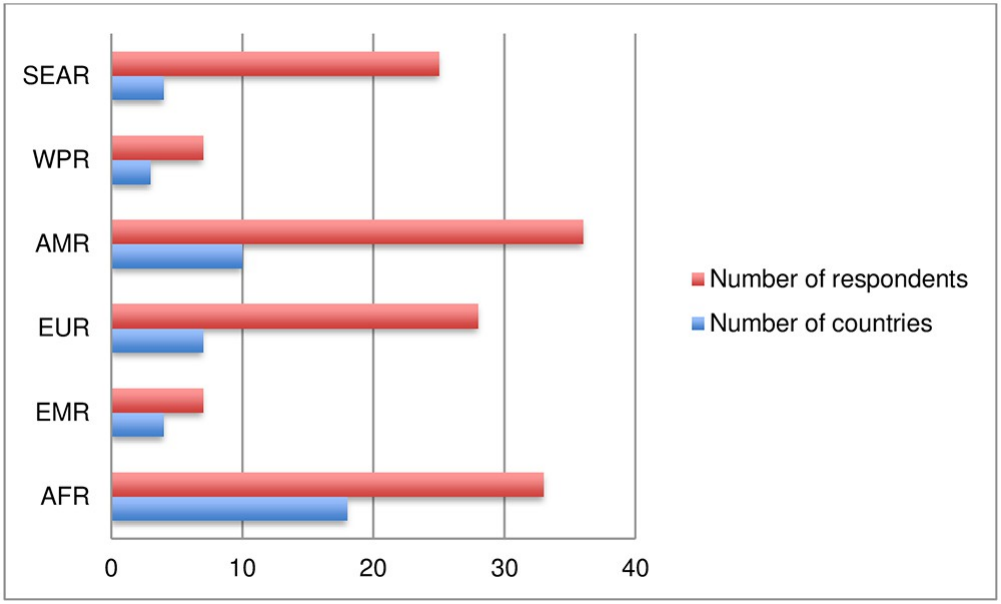
Number of countries and respondents by WHO region. AFR: African Region: AMR: Region of the Americas; EMR: Eastern Mediterranean Region; EUR: European Region; SEAR: South-East Asia Region; WPR: Western Pacific Region.

Of the 318 people who attempted to participate in the online survey, 142 provided complete answers and 138 declared their country. The survey respondents were from 47 countries, from all continents: North and sub-Saharan Africa, Asia, Europe, Latin America, Middle East, North America and Oceania (Fig 1). The geographical distribution of respondents indicates that 26% (36) were from North and sub-Saharan Africa from 20 countries, 25% (35) from Latin America from 9 countries, 22% (31) from Asia from 6 countries, 20% (28) of respondents were from 7 countries in Europe. The analysis of the survey respondents and their countries by WHO region shows that the highest number of respondents were from the Region of the Americas, followed by the African, European and South-East Asian regions. In terms of national coverage, the African Region was the most represented region, followed by the Region of the Americas (Fig 2).

**Fig 3:**
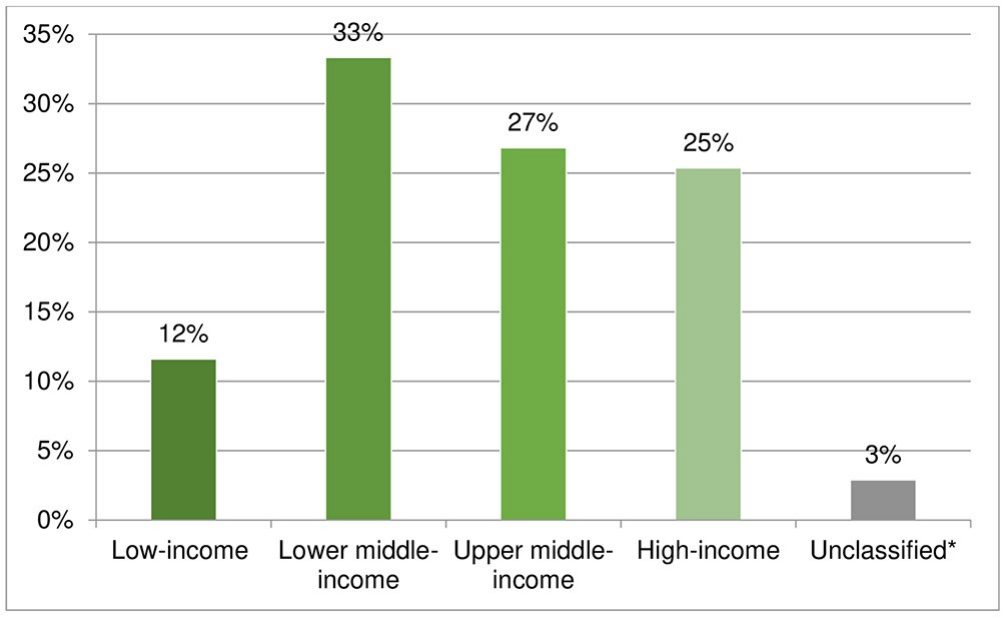
Indication of country level income for respondents. *Source*: New World Bank country classifications by income level: 2022–2023 (https://blogs.worldbank.org/opendata/new-world-bank-country-classifications-income-level-2022-2023).

Most respondents were from upper and lower middle-income countries (60%), hence the purpose of the survey to provide information on the level of drug repurposing prescribed in middle-income countries was achieved. There were also 35 (25%) respondents from 12 high-income countries, while only 16 (8%) respondents were from low-income countries (Fig 3).

**Fig 4:**
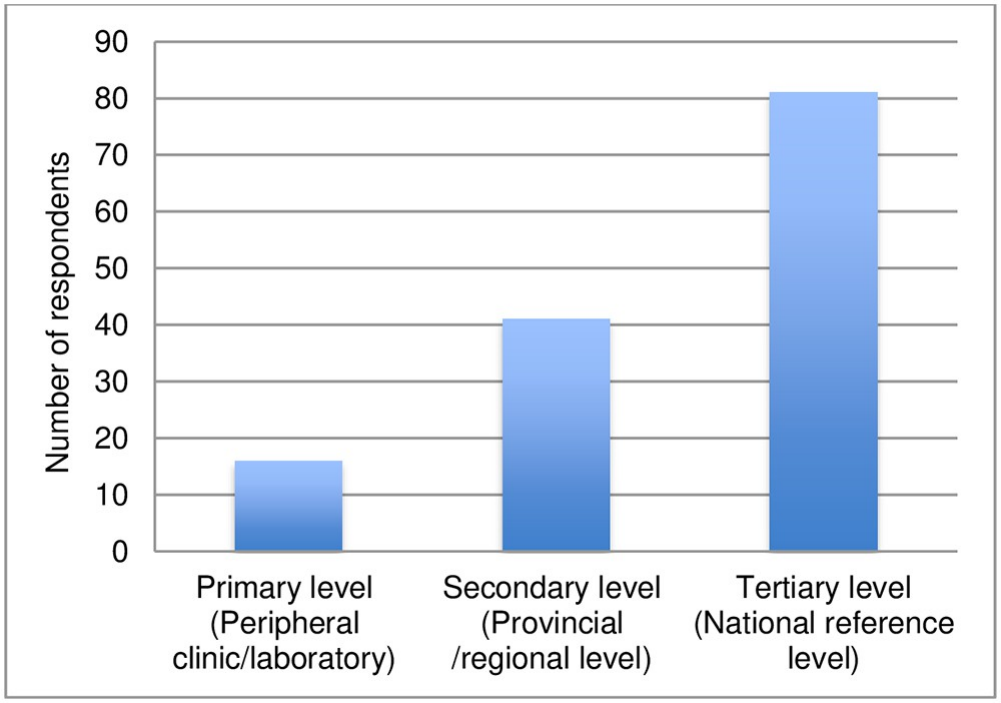
Respondents by health system setting.

**Fig 5:**
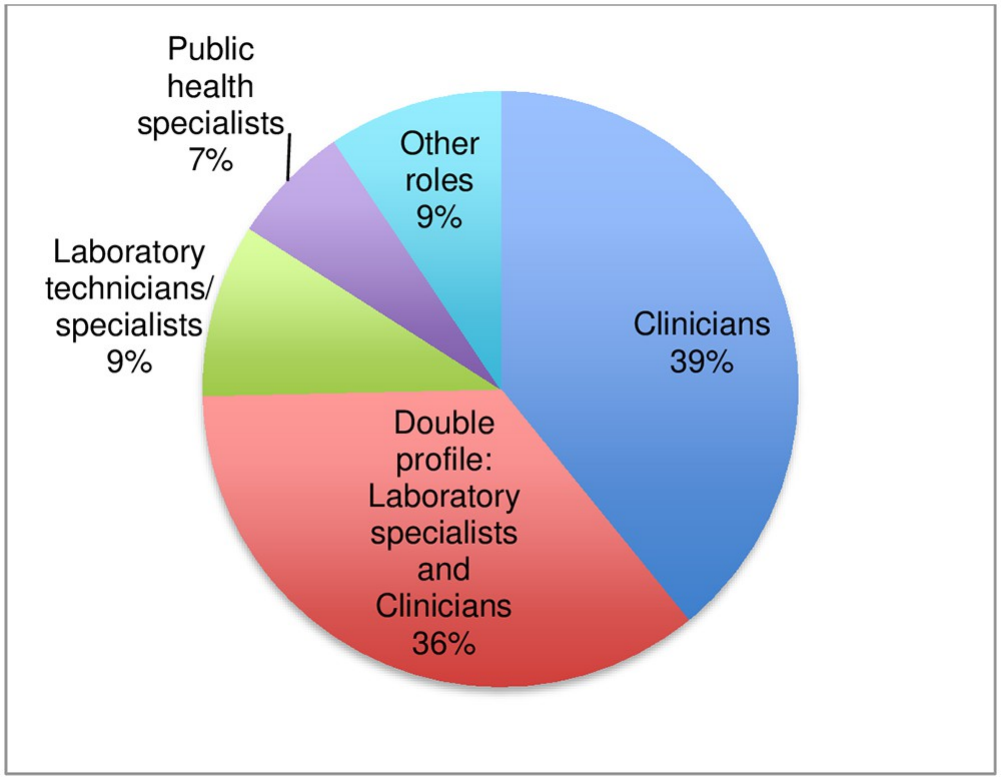
Respondents by professional role.

Of the 142 respondents, 138 disclosed their work settings: 59% (81) reported working at the tertiary level (national reference level), 30% (41) worked at the secondary level (at provincial/regional level) and 11% (16) reported working at the primary level (peripheral clinic or laboratory) (Fig 4). Also, 138 respondents disclosed their professional role: 39% (54) categorized themselves as clinicians, 36% (49) as having a double profile as laboratory specialists and clinicians, 9% (13) as laboratory technicians/specialists, 7% (9) as public health specialists, and 9% (13) categorized themselves as having other roles (pharmacist, mycologist, researcher, consultant, professor) (Fig 5).

### Diagnostic techniques

**Fig 6:**
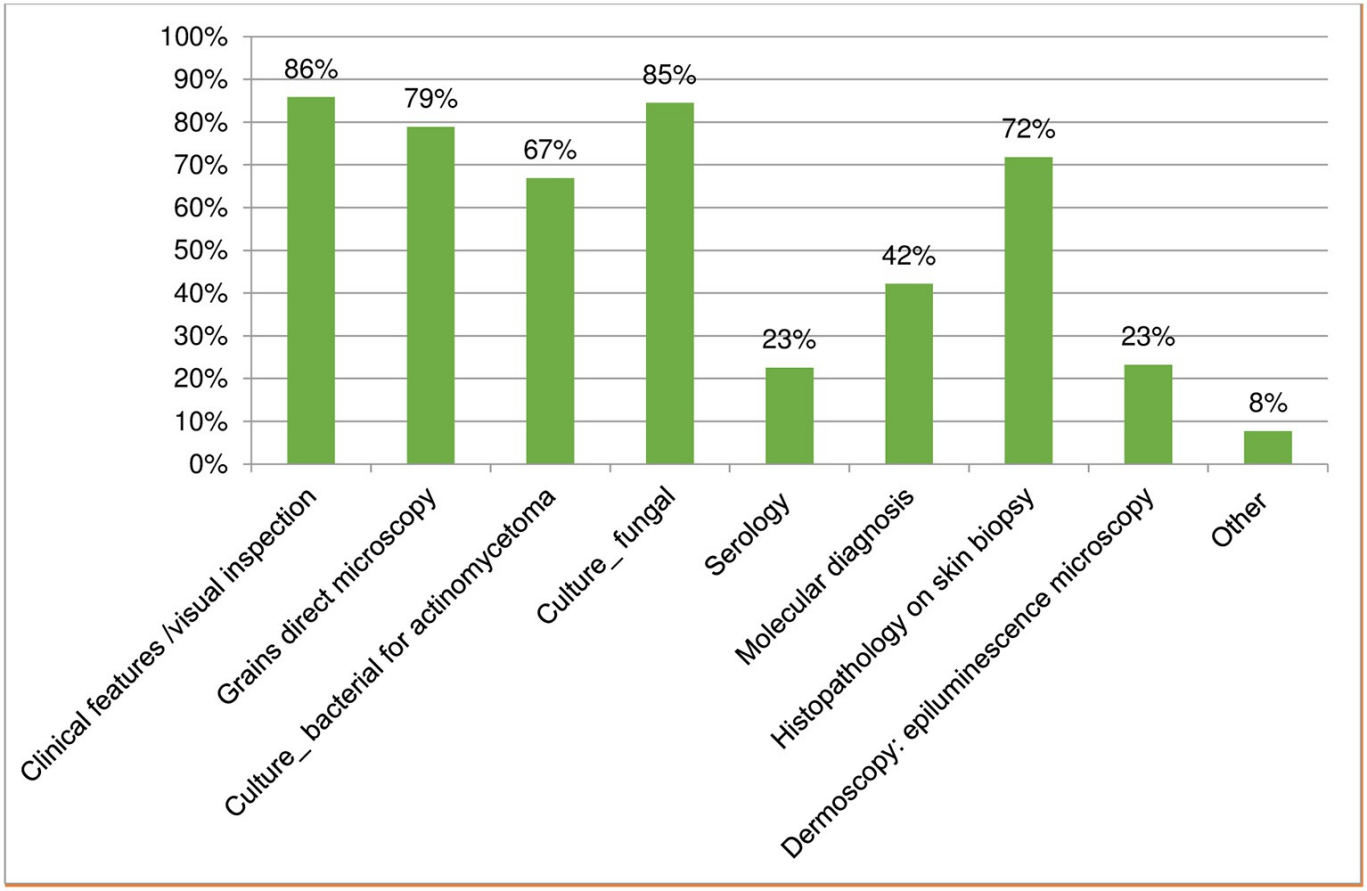
Diagnostic methods available for implantation mycoses indicated by respondents.

The respondents provided data on the diagnostic techniques available in their setting for the diagnosis of implantation mycoses: 86% (122) used clinical features/visual inspection; grains direct microscopy and histopathology on skin biopsy was available for 79% (112) and 72% (102) of respondents, respectively. Bacterial culture for actinomycetoma was available to 67% (95) of respondents, while fungal culture was available to 85% (120) of respondents. Molecular diagnosis was reported to be available to 42% (60), while serology was available to 23% (32). Dermoscopy: epiluminescence microscopy was reported as available for 23% (33) of respondents. Of the other techniques not listed, matrix-assisted laser desorption ionization time-of-flight (MALDI-TOF) was indicated by four respondents (Fig 6).

A sub-analysis on diagnostic methods available by health system level was performed to consider trends (Table 1). It shows that several diagnostic methods for implantation mycoses were available at tertiary and secondary levels with similar trends. At the primary level, there was a generalized trend for lower availability of diagnostic techniques other than visual inspection. Grains direct microscopy was available only at 56%, bacterial culture for actinomycetoma available at 38%, molecular diagnosis and histopathology on skin biopsy at 31%, and epiluminescence microscopy at 13% of primary level sites. Serology was only limitedly available and used, independent of health system level, for the diagnosis of implantation mycoses (21–27% of sites).

**Table 1:** Disaggregated data on diagnostic methods available by level of the health system.

Diagnostic methods	Tertiary level		Secondary level		Primary level	
	Indicated use by respondent (81)	Percentage	Indicated use by respondent (41)	Percentage	Indicated use by respondent (16)	Percentage
Clinical features/visual inspection	70	86%	38	93%	14	88%
Grains direct microscopy	66	81%	37	90%	9	56%
Bacterial culture for actinomycetoma	58	72%	31	76%	6	38%
Fungal culture	72	89%	36	88%	12	75%
Serology	17	21%	11	27%	4	25%
Molecular diagnosis	38	47%	17	41%	5	31%
Histopathology on skin biopsy	65	80%	32	78%	5	31%
Dermoscopy: epiluminescence microscopy	22	27%	9	22%	2	13%
Other	9	11%	2	5%	0	0%

### Eumycetoma: Medicines used for treatment and non-pharmacological interventions

**Fig 7:**
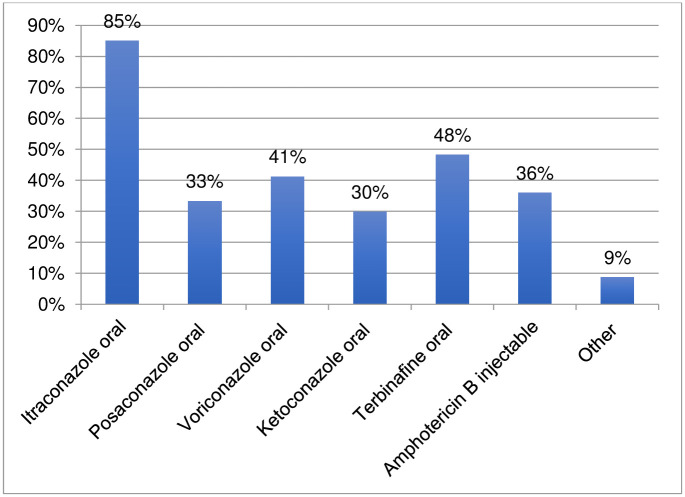
Medicines used to treat eumycetoma.

One hundred fourteen respondents provided information regarding the medicines used to treat eumycetoma (Fig 7, S1 Table). Itraconazole was the most commonly used medicine (85%), followed by terbinafine (48%). The latest generation azoles (posaconazole and voriconazole) were used by 33% and 41% of respondents, respectively, mainly in high-income countries and certain middle-income countries. Surprisingly, oral ketoconazole is still used by 30% of respondents, despite it having been removed from the WHO Essential Medicines List in 2000 (as an antifungal medicine) and despite hepatotoxicity alerts issued by WHO and by Stringent Regulatory Authorities [10,11,12,13]. Injectable Amphotericin B was indicated as being used by 36% of respondents; one respondent also indicated the use of its liposomal form. Other medicines indicated by respondents were fluconazole, griseofulvin, isavuconazole, dapsone, and olorofim. The respondents’ comments related to the medicines used for eumycetoma indicated that itraconazole is very expensive and not always available. Also terbinafine, used for refractory cases, was indicated by a few respondents as expensive. Affordability was mentioned as one of factors in the choice of medicines in several instances in the comments. For example, fluconazole and ketoconazole were reported as used because they are more affordable than other antifungal azole agents.

**Fig 8:**
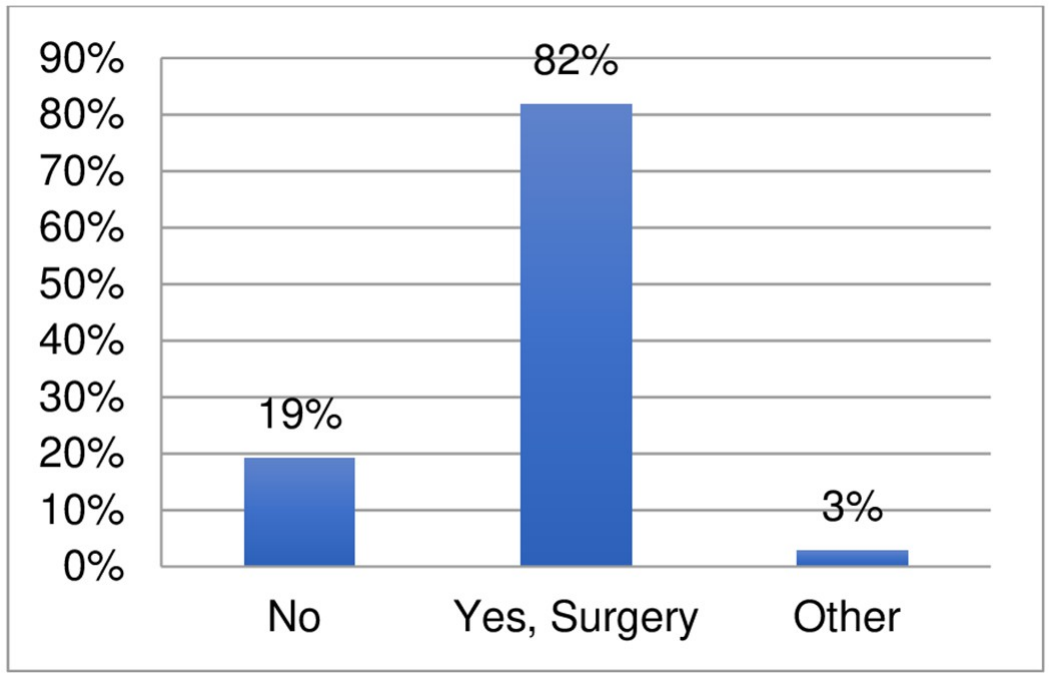
Non-pharmacological interventions applied for the treatment of eumycetoma.

The survey also collected data on non-pharmacological interventions used for eumycetoma (Fig 8, S2 Table). 110 respondents provided information: 82% (90) indicated that surgery is applied in their setting for the treatment of eumycetoma. Three respondents indicated the use of other non-pharmacological interventions: cryotherapy, hyperthermia, and debridement. A few respondents provided comments indicating that surgery was applied jointly with pharmacological treatment in difficult cases. Surgical excision was applied jointly (in 2–3 phases) during pharmacological treatment. Amputation was also mentioned in two comments for cases, which presented too late to treatment, with one comment indicating that amputation may not be resolutive.

### Actinomycetoma: Medicines used for treatment

**Fig 9:**
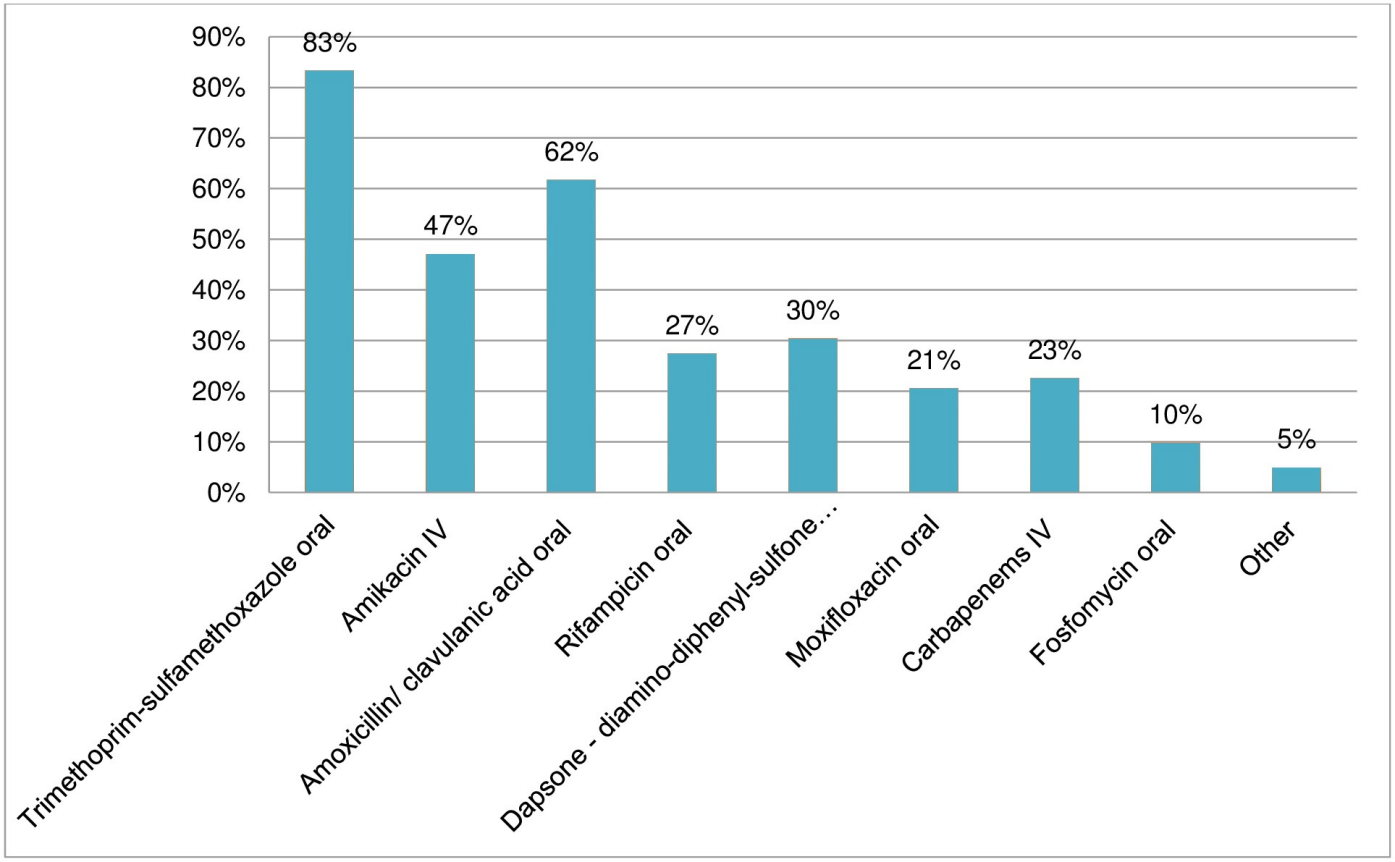
Medicines used to treat actinomycetoma.

One hundred two respondents provided information regarding the medicines used for actinomycetoma (Fig 9, S3 Table). The medicines most used to treat this bacterial-caused infection include trimethoprim–sulfamethoxazole (83%) and amoxicillin/clavulanic acid (62%). Amikacin injectable was used by 47% of respondents; dapsone and rifampicin were used respectively by 30% and 27% of respondents. There was also a considerable use of injectable carbapenems (23%), moxifloxacin (21%) and fosfomycin (10%). Among other medicines used for actinomycetoma, five respondents indicated use of levofloxacin, clindamycin, gentamicin injectable, doxycycline, rifampicin and isoniazid. Of the 12 respondents who provided comments, three indicated that the drug combination or choice of medicines was based on culture strain identification or other diagnostic identification techniques (microscopy, culture, polymerase chain reaction) and drug susceptibility testing. One comment indicated that treatment is tailored to the type of patient and his or her commitment to treatment. A few comments related to the affordability and availability of medicines. The availability of dapsone and rifampicin was also mentioned as linked to disease-specific treatments (leprosy multidrug therapy and antituberculosis regimens, respectively). Two comments indicated the toxicity of amikacin for massive lesions and/or long treatment duration (6–12 months). The use of amikacin, despite its ototoxicity and nephrotoxicity, most likely reflects the adoption and adaptations of the “Welsh regimen”, a combination of trimethoprim–sulfamethoxazole oral with injectable amikacin in one to four-week cycles for use in cases refractory or resistant to previous treatment and/or with involvement of underlying organs or bone [14, 15].

### Chromoblastomycosis: Medicines used for treatment, non-pharmacological interventions, and refractory cases

**Fig 10:**
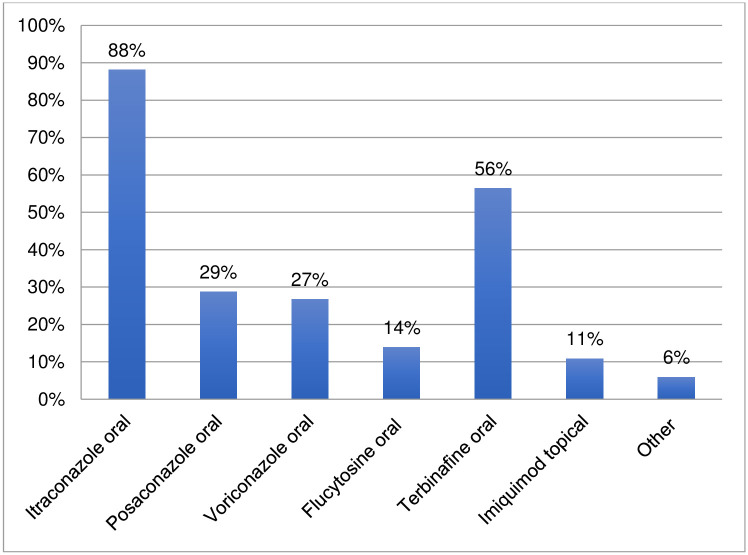
Medicines used to treat chromoblastomycosis.

One hundred one respondents provided information regarding the medicines used for chromoblastomycosis (Fig 10, S4 Table). Itraconazole was the most frequently reported medicine used to treat chromoblastomycosis (88%), followed by terbinafine (56%); posaconazole (29%) and voriconazole (27%). The respondents also reported a lower, but still substantial, use of flucytosine (14%) and topical imiquimod (11%). Another five medicines were listed by respondents as being used: potassium iodide, isavuconazole, fluconazole, topical fluorouracil, and injectable amphotericin B. Comments from approximately 25 respondents reported that often chromoblastomycosis pharmacological treatment is combined with non-pharmacological interventions, depending on the gravity and extension of the lesions.

**Fig 11:**
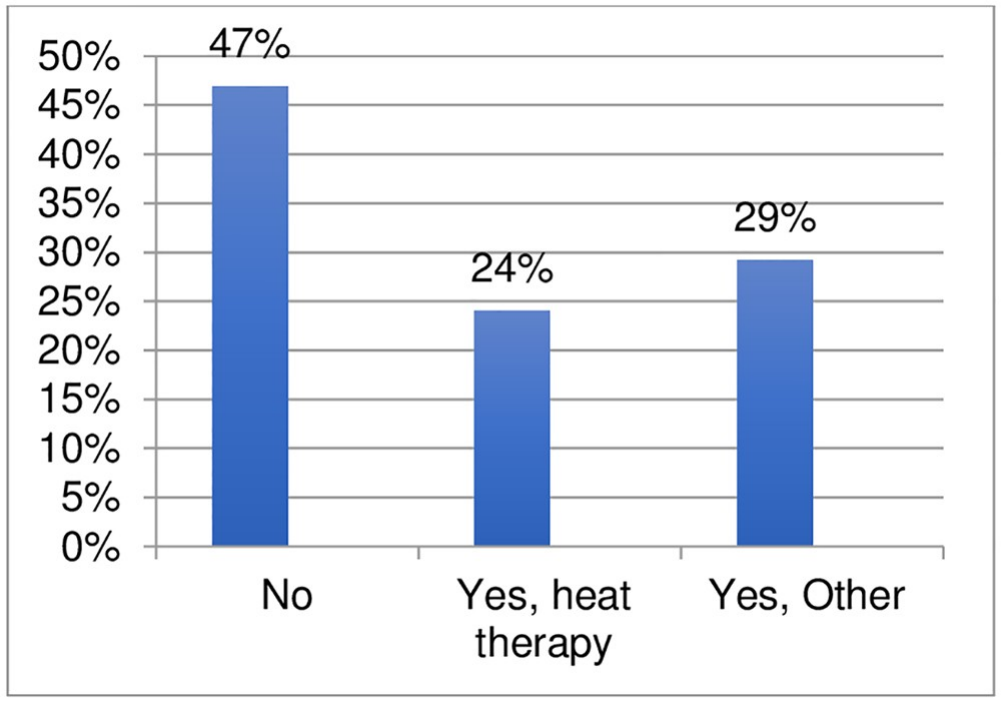
Non-pharmacological interventions applied for the treatment of chromoblastomycosis.

**Fig 12:**
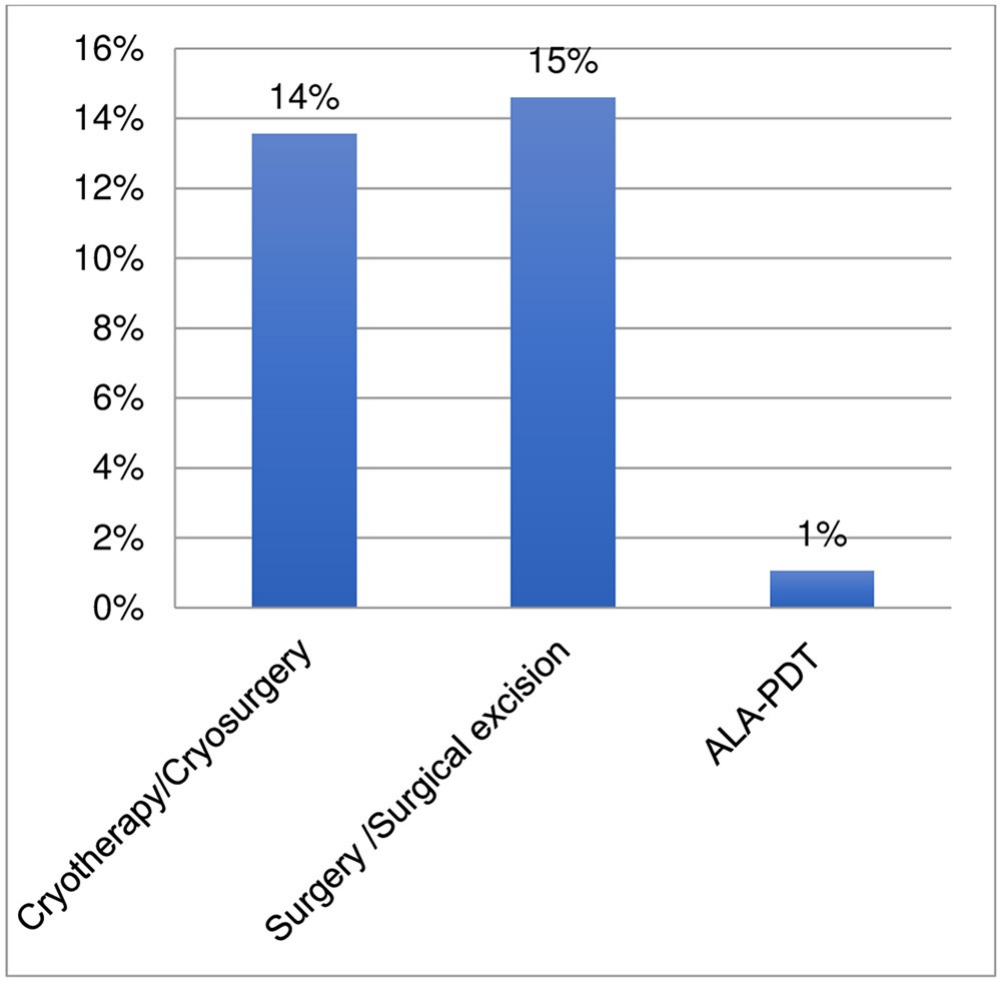
Disaggregated data for other non-pharmacological interventions indicated for the treatment of chromoblastomycosis.

Ninety-six respondents provided information regarding non-pharmacological treatments used for chromoblastomycosis (Figs 11 and 12, S5 Table). 53% of respondents indicated the use of non-pharmacological interventions including: heat therapy (24%), cryotherapy/cryosurgery (14%), and surgery/surgical excision (15%). One respondent also indicated the use of 5-aminolevulinic acid photodynamic therapy (ALA-PDT). The comments on the use of non-pharmacological interventions (in particular, cryotherapy) indicate that they are used in combination with the most used medicines: itraconazole and/or terbinafine. In selected cases, cryotherapy is used in combination with pharmacological treatment, instead of increasing the medicine dosage. One comment suggested that use of non-pharmacological methods should be considered since itraconazole and terbinafine are unavailable and too expensive. Two comments indicated that some patients with a long treatment history and large lesions may need surgery as adjuvant therapy.

Eighty-three respondents provided information regarding treatment of refractory cases; 68% reported refractory cases of chromoblastomycosis in their setting (S6 Table). Comments related to refractory cases suggest that they are caused by disease severity, late diagnosis, and interruptions of treatment (also due to cost and length), which result in increased severity of the lesions and relapses. Refractory cases do not seem linked to antifungal resistance as per the provided comments.

### Cutaneous sporotrichosis: Medicines used for treatment and refractory cases

**Fig 13:**
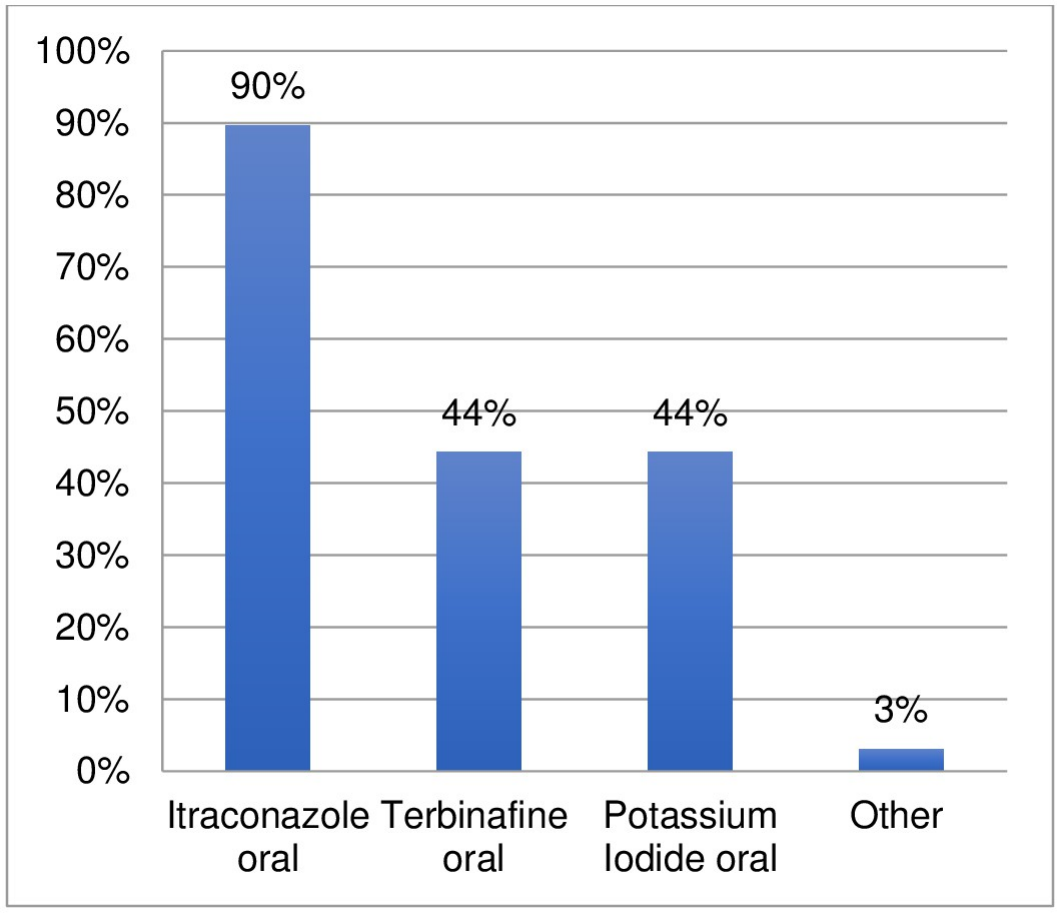
Medicines used to treat cutaneous sporotrichosis.

Ninety-seven respondents provided information regarding the medicines used for cutaneous sporotrichosis (Fig 13, S7 Table). Ninety percent reported using itraconazole and indicated considerable use of both terbinafine (44%) and potassium iodide (44%). Another four medicines were listed by three respondents: fluconazole, voriconazole, posaconazole, and injectable liposomal amphotericin B. The comments provided related to different aspects including geographical distribution of the disease, therapeutic protocols, as well as availability and affordability of medicines.

Eighty-seven respondents also provided information regarding refractory cases; 34% reported refractory cases of cutaneous sporotrichosis in their setting (S8 Table). From the provided comments, it seems that for cutaneous sporotrichosis, some cases are refractory due to interruption of treatment (high cost/long duration) or late diagnosis. Potassium iodide and terbinafine were indicated as used for treatment of refractory cases, alone or in combination with first-line itraconazole (at higher dosage). One comment also referred to heat therapy use for refractory cases.

### Availability and affordability of medicines

**Fig 14:**
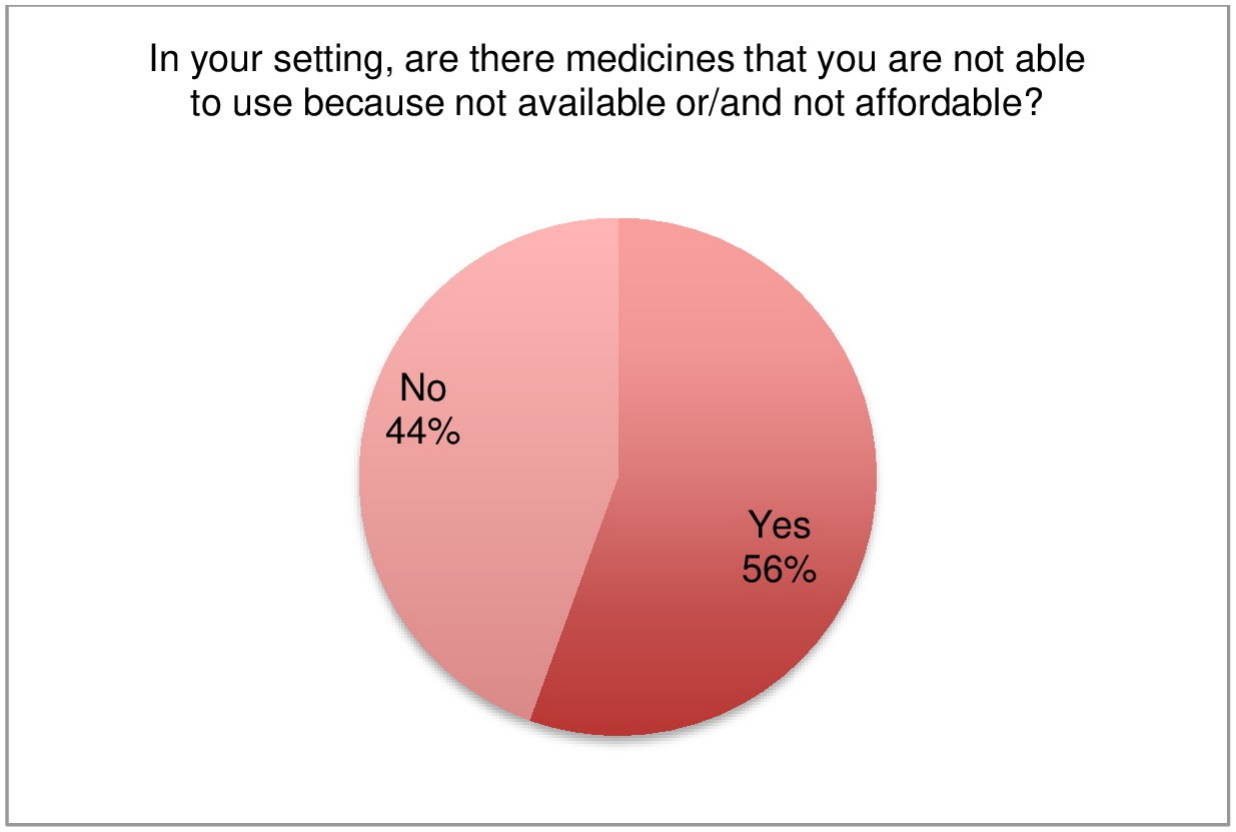
Availability and/or affordability of medicines in respondents’ settings.

One hundred thirty-five respondents answered the question related to availability and/or affordability of medicines for treatment of implantation mycoses; 56% of respondents indicated that medicines were not available and/or affordable in their setting (Fig 14, S9 Table). The question as formulated in the survey did not provide an option for respondents to distinguish between availability and affordability of medicines. The collected data combines both concepts related to access to medicines. The main purpose of this question was to determine whether there were barriers to accessing and using medicines and their repurposing due to lack of availability or unaffordability of medicines in countries. The survey confirms that use of medicines, and therefore drug repurposing, is influenced by the availability and/or affordability of medicines in countries.

A sub-analysis by country income level that was performed shows respondents indicating lack of availability and/or affordability of medicines to treat implantation mycoses resides, as expected, in low-income countries where incapacity to use medicines is reported to be 93% (S10 Table). With the exception of respondents from high-income countries, respondents from lower and upper middle-income countries reported that access to recommended medicines was a major problem.

The respondents also indicated which medicines are not available and/or affordable in their setting (Table 2). In decreasing order, the most unavailable and/or unaffordable medicines were: posaconazole, voriconazole, itraconazole, liposomal amphotericin B, flucytosine, amphotericin B, dapsone, terbinafine, potassium iodide, rifampicin. Interestingly, itraconazole, which is empirically considered the first-line treatment for eumycetoma, chromoblastomycosis and sporotrichosis–is among the medicines most listed as not available and/or affordable, following second-generation azoles (posaconazole, voriconazole). Availability/affordability is reported as a problem also for flucytosine, terbinafine, amphotericin B (including the liposomal form) and potassium iodide. Several medicines used for treatment of actinomycetoma have also been listed and reported as challenging to access (dapsone, amikacin, streptomycin, carbapenems, rifampicin). Hence, the level of drug repurposing is also limited by restricted availability and/or affordability of medicines in countries. Moreover, this seems to affect outcomes of treatment as several comments through the survey indicated that even first-line medicines may not be available and/or affordable to patients (not included in health insurance schemes, out-of-pocket purchase by patients, unavailability in the country).

**Table 2:** Medicines indicated as unavailable and/or unaffordable in the open comment question.

Medicine indicated as unavailable and/or unaffordable	No. of respondents
Posaconazole oral	24
Voriconazole oral	23
Itraconazole oral	17
Flucytosine oral	7
Liposomal amphotericin B injectable	7
Amphotericin B injectable	5
Dapsone oral	5
Terbinafine oral	4
Amikacin injectable	4
Streptomycin injectable	3
Potassium iodide oral	2
Rifampicin oral	1
Carbapenems injectable	1
Imiquimod topical	1

### Other considerations provided on access to treatment by the survey respondents

The survey allowed the respondents to provide comments for each question. Several respondents highlighted that access to treatment is a major problem in their setting. Most patients do not have the economic means to pay for treatment, either because they are low-income and/or are living in “extreme poverty”. Patients have to pay out of pocket (either partially or totally) for treatment, which is a major barrier to complying with a treatment that is often long. More than one respondent indicated the choice of suboptimal prescribed treatment, in order to make it affordable to patients. The economic barriers affect the capacity of patients to comply with the prescribed treatment and, in certain settings, even to afford the transportation costs to come to the medical consultation.

Price barriers may also affect patients with “imported cases” (i.e., migrants from endemic countries) in high-income countries, as reported by two respondents. National health insurance schemes in such countries may not recognize treatment for implantation mycoses (due to lack of disease classification within the health system). In high-income countries where private insurance schemes are used, migrants may not be insured. One comment suggests that not only cost, but also procurement of certain medicines, may be challenging in high-income countries for treatment of “imported cases”.

Treatment capacity to prescribe medicines is linked to availability of diagnostic methods. Several respondents highlighted diagnostic capacity as a major problem in correctly diagnosing and prescribing medicines for implantation mycoses. The need for training of health professionals and clinicians was also pointed out by respondents. The inclusion of medicines in the List of Essential Medicines was also called upon jointly with a request to deliver these medicines free of charge to patients and make medicines available and affordable to low-and middle-income countries. A few comments called for the development of treatment guidelines for all implantation mycoses. Comments also suggested that in certain middle-income countries these diseases are not recognized or prioritized by governments and health ministries, hence the lack of procurement and supply schemes for free treatment applied for other diseases.

## Discussion

The main findings should be considered in light of the respondents’ profiles: 85% were from high- and middle-income countries. Although with several limitations, the survey provides interesting indications on diagnostic capacity, treatment trends, and medicines’ availability, as well as challenges for implantation mycoses globally, especially in middle-income countries. These findings could prompt additional and more in-depth investigations to inform strategies and actions of implementing a road map for implantation mycoses.

The first survey, which attempted to determine the burden of disease for mycetoma, was performed by WHO between December 2016 and April 2017 [16]. As reported in the road map, as of 2020 only 1 out of 30 countries had a surveillance system in place for mycetoma. The spontaneous collection of case reports through an openly accessible platform could support collection of observational real-world data on causative agents, diagnostic methods, medicines and, where applicable, non-pharmacological interventions used for these diseases. It could also allow reporting and analysing treatment outcomes, possibly with an indication of the severity of the disease at patient enrolment. One of the main objectives of the road map is to identify shorter, more effective treatments for this group of diseases, in addition to early detection through point-of-care or near point-of-care diagnostic methods. Treatment efficacy for implantation mycoses is low. As an example, for actinomycetoma the cure rate with antibiotics is 60–90%, and for eumycetoma with antifungals is generally low (< 30%) [5,6].

While one phase II proof-of-concept trial to test a new azole, fosravuconazole, for the treatment of eumycetoma by the Drugs for Neglected Diseases *initiative* (DNDi) and the WHO Collaborating Centre on Mycetoma in Sudan (the Mycetoma Research Centre of the University of Khartoum) has been concluded in 2022, there are no other clinical trials evaluating new chemical entities for these diseases [17]. There is one registered study, a single arm observational study for the use of a non-pharmacological intervention in chromoblastomycosis (5-aminolevulinic acid photodynamic therapy (ALA-PDT)) at the Southern Medical University in Guangzhou, Guangdong, China [18]. The research and development pipeline for new treatments for implantation mycoses is basically empty, although there are ongoing efforts focused on identifying new chemical entities with new modes of action using an open-source drug discovery approach [6,19,20]. Several promising compounds and classes have been identified with the azoles and diiodohydroxyquinoline (iodoquinol) being the only two classes of compounds active against all the causative agents of all three fungal implantation diseases: eumycetoma, chromoblastomycosis, and sporotrichosis [21]. The study indicated that among the azoles, ravuconazole and posaconazole showed the strongest activity against the causative agents of all three fungal skin NTDs [6]. According to preliminary results presented by the Mycetoma Research Centre in Sudan at the 21st Congress of the International Society for Human and Animal mycology in 2022, fosravuconazole did not show superiority over the current standard treatment itraconazole in the clinical trial run at the centre supported by DNDi.

There are currently no registered clinical trials of fosravuconazole, the prodrug of ravuconazole, for chromoblastomycoses and sporotrichosis. Similarly, clinical studies for other antifungal medicines and which have been found to be promising for implantation mycoses such as olorofim are lacking [22,23]. Echoing the Khartoum call for action on mycetoma and expanding it to chromoblastomycosis and sporotrichosis, researchers, clinicians, and members of the international community and pharmaceutical companies are requested to promote consensus towards public health strategies and encourage collaborative research and development with a coordinated approach and building on existing initiatives [24]. In the move to a more integrated approach to identify medicines active against all three fungal skin NTDs, compounds made in the MycetOS initiative, created by DNDi for identification of compounds active for mycetoma, could also be explored systematically for chromoblastomycosis and sporotrichosis with a view towards integrated and coordinated preclinical and clinical research [6].

It shall be noted that there are no WHO evidence-based guidelines or treatment recommendations for implantation mycoses. For these diseases, the evidence referred to as ideal “gold standard” for the development of treatment guidelines (the systematic review of clinical trials) is absent. As inferred by the WHO-adopted GRADE methodology and by the WHO Guidelines Review Committee procedures, the collection of evidence in the absence of clinical trials (as is the case for implantation mycoses) would need to rely on the systematic review of non-randomized trials and observational studies, including case series and case reports [25]. Institutions such as the European Confederation of Medical Mycology (ECMM) in cooperation with the International Society for Human and Animal Mycology (ISHAM) have started issuing diagnosis and treatment guidelines on endemic mycoses using the GRADE methodology which would be helpful guidance for the endemic countries in the absence of WHO guidelines [26]. This effort, which is now covering only sporotrichosis as an implantation mycosis, may also be extended to chromoblastomycosis and eumycetoma for supporting WHO guidelines development. In addition to the use of the GRADE methodology, WHO follows a rigorous process in the development of guidelines and recommendations with a strong attempt to be inclusive and to assess and avoid any potential conflict of interest.

In the context of scarce evidence and potential for drug repurposing, the use of a readily accessible platform where clinicians could input case report information for implantation mycoses would constitute an important resource as a global treatment registry. The use of the openly accessible, publicly-funded CURE ID platform (https://cure.ncats.io) would also serve to collect the data in a consistent and structured manner, enabling quality information to be gathered, curated, and shared to facilitate aggregation of the case experiences.

The survey confirms that drug repurposing is occurring for eumycetoma, chromoblastomycosis and cutaneous sporotrichosis beyond itraconazole as first choice (85–90%) and terbinafine for refractory cases (44–56%). For eumycetoma and chromoblastomycosis, newer generation azoles (posaconazole and voriconazole) were also reported to have been used (27–41%). Older generation azoles are reportedly still used for eumycetoma (oral ketoconazole at 30%) and for chromoblastomycosis, with comments indicating that their use is linked to lower cost in relation to other azoles. Amphotericin B injectable is used for eumycetoma (36%). For chromoblastomycosis, there is a lower but consistent use of flucytosine (14%) and topical imiquimod (11%). For cutaneous sporotrichosis, a considerable use of oral potassium iodide is reported (44%).

For actinomycetoma, several antibiotics from several classes are used in oral and injectable forms, suggesting a high level of drug repurposing. The survey confirms the use of several survey listed medicines (ranging from 83% to 10%), and considerable use of injectable medicines (amikacin at 47%, carbapenems at 22%) indicating that there is no consensus on the optimal treatment across the endemic countries [15].

The survey also investigated the use of non-pharmacological interventions for eumycetoma and chromoblastomycosis. The survey confirms that surgery (and very rarely cryotherapy and/or hyperthermia) is applied in the treatment of eumycetoma in over 80% of settings. Nevertheless, the extent and modality of surgical applications for eumycetoma cannot be defined through the survey and it shall be investigated further. The survey confirms that non-pharmacological interventions are applied for treatment of chromoblastomycosis by 53% of respondents; these include heat therapy (24%), cryotherapy/cryosurgery (14%) and surgery (15%). The survey respondents reported a very high percentage of refractory cases of chromoblastomycosis (nearly 70%) with comments indicating that these cases are caused primarily by late diagnosis and interruption of treatment. The survey also indicated that cutaneous sporotrichosis has a considerable rate of refractory cases (34%). The high rates of refractory cases found by the survey calls for increased capacity to detect cases early at the point-of-care and treat them with shorter, more effective and affordable regimens.

The collection of data (both retrospective and prospective) on treatment combinations, up-front drug susceptibility testing where available, and identifying the etiological agent may help inform better treatment outcomes and could constitute observational data to inform treatment guidelines and clinical research. The collection of case report data should include both pharmacological treatment and non-pharmacological treatment, as the survey indicated that a spectrum of non-pharmacological interventions are applied in combination with pharmacological treatment. The application length, frequency, and type of non-pharmacological intervention (heat therapy, cryotherapy/cryosurgery, surgery) used should be also duly registered in case report registries.

The pilot testing and use of the CURE ID platform, supported by the implantation mycoses community of practice, could offer a repository of data on case reports in the form of a treatment registry that could be of significant value to identify candidates for additional research, encourage further drug development, inform the development of treatment guidelines and serve as a resource for treating physicians, given the dearth and challenge of conducting randomized controlled trials. In the context of implantation mycoses, the low prevalence and the diversity of etiological agents limit the ability to conduct these clinical trials to determine the effective and shorter treatment for these diseases.

Further, the survey findings suggest that evidence-based guidelines for these diseases are urgently needed and should address the entire spectrum of pharmacological and non-pharmacological interventions used for implantation mycoses. The highlighted challenges of availability and affordability of medicines also represent a constraint for the choice of effective treatment and for drug repurposing. Barriers to accessing medicines to treat implantation mycoses require further investigation and should be considered while prompting and using the generated real-world data to support clinical research hypotheses for drug repurposing. Policies and procurement schemes to ensure access to medicines should be an integral part of global and national actions to tackle this new group of neglected diseases.

## Supporting information

S1 Table Medicines used to treat eumycetoma. (DOCX)

S2 Table Non-pharmacological interventions applied for the treatment of eumycetoma. (DOCX)

S3 Table Medicines used to treat actinomycetoma. (DOCX)

S4 Table Medicines used to treat chromoblastomycosis. (DOCX)

S5 Table Non-pharmacological interventions applied for the treatment of chromoblastomycosis. (DOCX)

S6 Table Refractory cases of chromoblastomycosis. (DOCX)

S7 Table Medicines used to treat cutaneous sporotrichosis. (DOCX)

S8 Table Refractory cases of cutaneous sporotrichosis. (DOCX)

S9 Table Availability and/or affordability of medicines in respondents’ settings. (DOCX)

S10 Table Stratification of respondents by income country level indicating non availability and/or affordability of medicines. * Bolivarian Republic of Venezuela.
(DOCX)
