# Supplementary material for: Diagnostic capacities and treatment practices on implantation mycoses: Results from the 2022 WHO global online survey
Source: PLoS Negl Trop Dis. 2023 Jun 28;17(6):e0011443. doi: 10.1371/journal.pntd.0011443 (PMC10335693; doi:10.1371/journal.pntd.0011443)
Supplement: S1 Table — (DOCX) [file pntd.0011443.s001.docx]

**S1 Table. Medicines used to treat eumycetoma**

| **Medicine** | **Indicated use by respondent (114)** | **Percentage** |
| --- | --- | --- |
| Itraconazole oral | 97 | 85% |
| Posaconazole oral | 38 | 33% |
| Voriconazole oral | 47 | 41% |
| Ketoconazole oral | 34 | 30% |
| Terbinafine oral | 55 | 48% |
| Amphotericin B injectable | 41 | 36% |
| Other | 10 | 9% |
| - Fluconazole oral (3) |  |  |
| - Griseofulvin oral (2) |  |  |
| - Liposomal amphotericin B injectable |  |  |
| - Isavuconazole oral |  |  |
| - Dapsone oral |  |  |
| - Olorofim oral |  |  |
|  |  |  |
