# Supplementary material for: Diagnostic capacities and treatment practices on implantation mycoses: Results from the 2022 WHO global online survey
Source: PLoS Negl Trop Dis. 2023 Jun 28;17(6):e0011443. doi: 10.1371/journal.pntd.0011443 (PMC10335693; doi:10.1371/journal.pntd.0011443)
Supplement: S2 Table — (DOCX) [file pntd.0011443.s002.docx]

**S2 Table. Non-pharmacological interventions applied for the treatment of eumycetoma**

| **Answer** | **Indicated use by respondent (110)** | **Percentage** |
| --- | --- | --- |
| None | 21 | 19% |
| Surgery | 90 | 82% |
| Other | 3 | 3% |
| - Hyperthermia and/or cryotherapy |  |  |
| - Cryotherapy |  |  |
| - Wound care, debridement |  |  |
