# Supplementary material for: Diagnostic capacities and treatment practices on implantation mycoses: Results from the 2022 WHO global online survey
Source: PLoS Negl Trop Dis. 2023 Jun 28;17(6):e0011443. doi: 10.1371/journal.pntd.0011443 (PMC10335693; doi:10.1371/journal.pntd.0011443)
Supplement: S3 Table — (DOCX) [file pntd.0011443.s003.docx]

**S3 Table. Medicines used to treat actinomycetoma**

| **Medicine** | **Indicated use by respondent (102)** | **Percentage** |
| --- | --- | --- |
| Trimethoprim + sulfamethoxazole oral | 85 | 83% |
| Amikacin injectable | 48 | 47% |
| Amoxicillin + clavulanic acid oral | 63 | 62% |
| Rifampicin oral | 28 | 27% |
| Dapsone - diamino-diphenyl-sulfone (DDS) oral | 31 | 30% |
| Moxifloxacin oral | 21 | 21% |
| Carbapenems injectable | 23 | 23% |
| Fosfomycin oral | 10 | 10% |
| Other | 5 | 5% |
| - Levofloxacin oral |  |  |
| - Clindamycin oral |  |  |
| - Gentamicin injectable |  |  |
| - Doxycycline oral |  |  |
| - Rifampicin oral |  |  |
| - Isoniazid oral |  |  |
