# Supplementary material for: Diagnostic capacities and treatment practices on implantation mycoses: Results from the 2022 WHO global online survey
Source: PLoS Negl Trop Dis. 2023 Jun 28;17(6):e0011443. doi: 10.1371/journal.pntd.0011443 (PMC10335693; doi:10.1371/journal.pntd.0011443)
Supplement: S4 Table — (DOCX) [file pntd.0011443.s004.docx]

**S4 Table. Medicines used to treat chromoblastomycosis**

| **Medicine** | **Indicated use by respondent (101)** | **Percentage** |
| --- | --- | --- |
| Itraconazole oral | 89 | 88% |
| Posaconazole oral | 29 | 29% |
| Voriconazole oral | 27 | 27% |
| Flucytosine oral | 14 | 14% |
| Terbinafine oral | 57 | 56% |
| Imiquimod topical | 11 | 11% |
| Other | 2 | 6% |
| - Potassium iodide oral |  |  |
