# Supplementary material for: Diagnostic capacities and treatment practices on implantation mycoses: Results from the 2022 WHO global online survey
Source: PLoS Negl Trop Dis. 2023 Jun 28;17(6):e0011443. doi: 10.1371/journal.pntd.0011443 (PMC10335693; doi:10.1371/journal.pntd.0011443)
Supplement: S5 Table — (DOCX) [file pntd.0011443.s005.docx]

**S5 Table. Non-pharmacological interventions applied for the treatment of chromoblastomycosis**

| **Answer** | **Indicated use by respondent (96)** | **Percentage** |
| --- | --- | --- |
| None | 45 | 47% |
| Heat therapy | 23 | 24% |
| Other | 28 | 29% |
| - Cryotherapy/cryosurgery | 13 | 14% |
| - Surgery/surgical excision | 14 | 15% |
| - 5-aminolevulinic acid photodynamic therapy (ALA-PDT) | 1 | 1% |
