# Supplementary material for: Diagnostic capacities and treatment practices on implantation mycoses: Results from the 2022 WHO global online survey
Source: PLoS Negl Trop Dis. 2023 Jun 28;17(6):e0011443. doi: 10.1371/journal.pntd.0011443 (PMC10335693; doi:10.1371/journal.pntd.0011443)
Supplement: S6 Table — (DOCX) [file pntd.0011443.s006.docx]

**S6 Table. Refractory cases of chromoblastomycosis**

| **Answer** | **Indicated by respondent (83)** | **Percentage** |
| --- | --- | --- |
| Yes | 56 | 68% |
| No | 26 | 32% |
