# Supplementary material for: Diagnostic capacities and treatment practices on implantation mycoses: Results from the 2022 WHO global online survey
Source: PLoS Negl Trop Dis. 2023 Jun 28;17(6):e0011443. doi: 10.1371/journal.pntd.0011443 (PMC10335693; doi:10.1371/journal.pntd.0011443)
Supplement: S7 Table — (DOCX) [file pntd.0011443.s007.docx]

**S7 Table. Medicines used to treat cutaneous sporotrichosis**

| **Medicines** | **Indicated use by respondent (97)** | **Percentage** |
| --- | --- | --- |
| Itraconazole oral | 87 | 90% |
| Terbinafine oral | 43 | 44% |
| Potassium iodide oral | 43 | 44% |
| Other | 3 | 3% |
| - Fluconazole oral |  |  |
| - Voriconazole and/or posaconazole oral (if itraconazole is not tolerated) |  |  |
| - Liposomal amphotericin B injectable |  |  |
