# Supplementary material for: Diagnostic capacities and treatment practices on implantation mycoses: Results from the 2022 WHO global online survey
Source: PLoS Negl Trop Dis. 2023 Jun 28;17(6):e0011443. doi: 10.1371/journal.pntd.0011443 (PMC10335693; doi:10.1371/journal.pntd.0011443)
Supplement: S9 Table — (DOCX) [file pntd.0011443.s009.docx]

**S9 Table. Availability and/or affordability of medicines in respondents’ settings**

| **Answer** | **Indicated use by respondent (135)** | **Percentage** |
| --- | --- | --- |
| Yes (not available and not affordable) | 75 | 56% |
| No | 60 | 44% |
