# Supplementary material for: Diagnostic capacities and treatment practices on implantation mycoses: Results from the 2022 WHO global online survey
Source: PLoS Negl Trop Dis. 2023 Jun 28;17(6):e0011443. doi: 10.1371/journal.pntd.0011443 (PMC10335693; doi:10.1371/journal.pntd.0011443)
Supplement: S10 Table — * Bolivarian Republic of Venezuela. (DOCX) [file pntd.0011443.s010.docx]

**S10 Table. Stratification of respondents by income country level indicating non availability and/or affordability of medicines**

| **Country income level countries** | **Number of respondents answering "Yes, not available/not affordable" (75)** | **Number of respondents answering the question (135)** | **Percentage** |
| --- | --- | --- | --- |
| Low-income countries | 14 | 15 | 93% |
| Lower middle-income countries | 30 | 47 | 64% |
| Upper middle-income countries | 26 | 34 | 76% |
| High-income countries | 1 | 35 | 3% |
| Unclassified * | 4 | 4 | 100% |

* Bolivarian Republic of Venezuela
